# Virtual Learning during COVID-19: Exploring Challenges and Identifying Highly Vulnerable Groups Based on Location

**DOI:** 10.3390/ijerph191711108

**Published:** 2022-09-05

**Authors:** Adi Jafar, Ramli Dollah, Ramzah Dambul, Prabhat Mittal, Syahruddin Awang Ahmad, Nordin Sakke, Mohammad Tahir Mapa, Eko Prayitno Joko, Oliver Valentine Eboy, Lindah Roziani Jamru, Andika Ab. Wahab

**Affiliations:** 1 Faculty of Social Sciences and Humanities, Universiti Malaysia Sabah, Kota Kinabalu 88400, Sabah, Malaysia; 2 Department of Commerce & Management, Satyawati College (Eve), University of Delhi, New Delhi 110021, India; 3 Institute of Malaysian and International Studies, Universiti Kebangsaan Malaysia (UKM), Bangi 43600, Selangor, Malaysia

**Keywords:** Sustainable Development Goals (SDGs), higher education, quality education, online learning, e-learning, Malaysia, Sabah

## Abstract

Amid the outbreak of the COVID-19 pandemic in the year 2020, educational platforms have been forced to change and adapt from conventional physical learning to virtual learning. Nearly all higher learning institutions worldwide are forced to follow the new educational setting through virtual platforms. Sabah is one of the poorest states in Malaysia with the poorest infrastructure, with the technology and communication facilities in the state remaining inept. With the changes in virtual platforms in all higher education institutions in Malaysia, higher learning institutions in Sabah are expected to follow the lead, despite the state lagging in its development. This has certainly impacted the overall productivity and performance of students in Sabah. Therefore, this study aims to explore the challenges of the implementation of virtual learning among students in Sabah. More specifically, this study seeks to identify vulnerable groups among students based on their geographical location. To achieve the objective of this study, a survey has been conducted on a total of 1,371 students in both private and public higher learning institutions in Sabah. The sample selection for this study was determined using a purposive sampling technique. Based on Principal Component Analysis (PCA), it was found that there are five challenges in virtual learning faced by students in higher learning institutions in Sabah. These are the unconducive learning environment (var(X) = 20.12%), the deterioration of physical health (var(X) = 13.40%), the decline of mental health (var(X) = 12.10%), the limited educational facilities (var(X) = 10.14%) and social isolation (var(X) = 7.47%). The K-Means Clustering analysis found that there are six student clusters in Sabah (Cluster A, B, C, D, E & F), each of which faces different challenges in participating in virtual learning. Based on the assessment of location, almost half of the total number of districts in Sabah are dominated by students from Cluster A (9 districts) and Cluster B (4 districts). More worryingly, both Cluster A and Cluster B are classified as highly vulnerable groups in relation to the implementation of virtual learning. The results of this study can be used by the local authorities and policymakers in Malaysia to improve the implementation of virtual learning in Sabah so that the education system can be more effective and systematic. Additionally, the improvement and empowerment of the learning environment are crucial to ensuring education is accessible and inclusive for all societies, in line with the fourth of the Sustainable Development Goals (SDG-4).

## 1. Introduction

Every individual on earth has the right to access a better and fair quality of educational opportunities regardless of their demographic, socio-economic status, and geographical location. This is in line with the fourth of the United Nations Sustainable Development Goal (SDG 4), which is to “ensure inclusive and equitable quality education and promote lifelong learning opportunities for all” [1]. The said education opportunities comprise pre-primary, primary, secondary, vocational, and tertiary levels, including university [2]. As stated in the ten goals of SDG 4, to achieve the quality of education, one of the requirements is to “Build and upgrade education facilities and provide safe, non-violent, inclusive and effective learning environments for all” [3], be it in the micro, meso or macro context [4]. 

In reality, there are many students in higher learning institutions that are still not able to access a fair and decent quality of education, particularly those populations in developing countries. Various aspects prevent the viability of educational settings so that they can be accessible to all populations. These include poor facilities in educational settings, weak teaching content, poor ICT knowledge, and poor network and communication infrastructures [5,6,7]. All these factors affect the viability and efficacy of learning environments. In the state of Sabah, where all these aforementioned problems exist, the situation became an educational crisis when Malaysia began to impose a national lockdown in March 2020 to control the spread of the pandemic of COVID-19 [8,9,10,11,12,13]. The pandemic of COVID-19 caused a global educational crisis [14], particularly in developing countries. Most developing countries such as Chile, India, Ecuador, Turkey, Mexico, Romania [15], China [16], and Malaysia [17,18] have transitioned their educational settings from physical learning to virtual learning, including in higher learning institutions [15,19], to break the transmission of COVID-19 [17,20]. In addition, the implementation of remote learning in the era of COVID-19 is an alternative to physical learning as a result of the extensive closure of schools and campuses during the period [21,22,23].

Unfortunately, there are several impediments to drastic changes in the educational environment from physical to virtual learning, as most developing countries were not fully prepared to implement comprehensive virtual learning, especially covering all the regions [24]. These include the lack of information and communications technology (ICT) infrastructure [24], electricity disruption and power outages [25], poor internet connection and access problems [26], poor study environments in following virtual learning at home [18] and low digital literacy among teachers and students [27]. In particular, virtual learning also poses health risks, both physically and mentally, when online teaching is conducted rigidly with regimented scheduling. Students have been shown to face health problems, such as headaches, obesity, body soreness, blurry vision, and eye fatigue [28], a decline in mental health with complications of stress, anxiety, and depression [29], decreasing sleeping quality [30], social isolation and lack of interaction [18,31,32], and declining academic achievements [33].

However, not all students demonstrate the same ability to participate in virtual learning activities, compared to those who could undergo it without problems. Moreover, this study posits that the more constraints faced by students in virtual learning, the higher the level of vulnerability faced in participating in the program, as demonstrated in the case of students in Sabah. In particular, this study argues that the level of vulnerability among students is broadly influenced by the environment at home and the geographical location. The existing study also found that there is a parallel relationship between a decent location and infrastructure and the delivery of virtual learning. Zhu et al., [34], for example, found that there is a digital divide between urban students and rural students in China. Almost half of the students in rural areas have not been able to access virtual learning due to limited ownership of electronic devices.

Almost all states in Malaysia, especially Sabah, are still lagging behind in the availability of educational facilities, such as poor internet access and limited ownership of electronic devices [35]. This condition should not be taken lightly, as it could lead to student dropouts [36]. A strategy of empowerment focusing on vulnerable students should be implemented following the fourth SDG to ensure inclusiveness for all individuals in getting educational opportunities, regardless of their geographical location and socioeconomic status. In the case of Malaysia, at present, there is no study on the condition of vulnerable groups among students that are badly affected by the national lockdown measures and COVID-19, regarding their experiences in virtual learning, particularly in Sabah. This illustrates the absence of empirical studies seeking to understand how students in higher institutions in Sabah are coping with the challenges of virtual learning. Moreover, with Sabah as the second largest state in Malaysia, with the poorest development, each of the district areas displays a different dynamic of challenges based on its location. Therefore, a study to identify the location of students in Sabah and the different dynamics of challenges based on these locations will certainly assist the Malaysian government to address the diverse needs of students depending on their district areas. This is to ensure that virtual learning in higher education can be executed more systematically and efficiently in Sabah. As such, this study aims to explore the challenges faced by students in higher education in Sabah during the implementation of virtual learning (known as P&P), and identify the vulnerable groups among students based on their residential location.

## 2. Methods

### 2.1. Study Area

**Figure 1:**
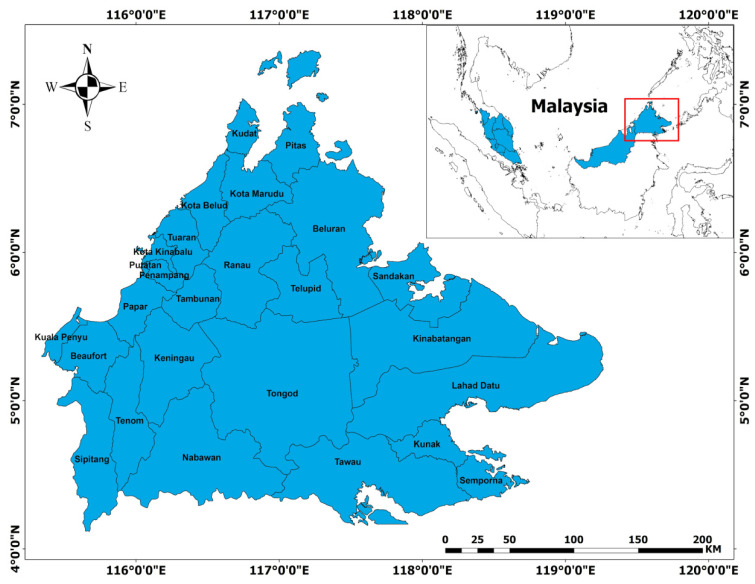
District position in Sabah, Malaysia.

Sabah is the second largest state in Malaysia with an area of 73,619 km^2^. The state of Sabah consists of five divisions, known as Sandakan (covering 28,205 km^2^), Tawau (14,905 km^2^), Interior (18,298 km^2^), West Coast (7588 km^2^), and Kudat (4623 km^2^). Each division contains a combination of several districts. For example, the Sandakan division has five districts, such as Kinabatangan, Beluran, Telupid, Sandakan and Tongod. Meanwhile, Lahad Datu, Tawau, Kalabakan, Kunak, and Semporna are located in the Tawau Division. The West Coast consists of Kota Belud, Kota Kinabalu, Penampang, Putatan, Papar, Ranau and Tuaran. Meanwhile, the Kudat division has the three districts Kota Marudu, Kudat, and Pitas. Both the West Coast and Kudat divisions have the highest and lowest number of districts, respectively. The Interior Division, on the other hand, has six districts, namely, Keningau, Tambunan, Beaufort, Kuala Penyu, Nabawan, Sipitang, and Tenom. In total, there are 27 districts in the state of Sabah, Malaysia, as indicated in Figure 1.

### 2.2. Data Collection

This study adopted a cross-sectional online survey among both postgraduate and undergraduate students in Sabah. The total sample of this study is 1371 students from three public universities and four private colleges in Sabah. According to Adam (2020) [37], for the continuous data category with a 99 percent confidence level, only 463 respondents are needed to represent a population with a value of infinity. Using Adam’s [37] as a standard requirement for sampling size, this study has already exceeded the minimum sampling size required to represent the study population. The sample selection for this study was determined using a purposive sampling technique. Eligibility criteria were determined using these selections; the sample consists of students in private and public higher education with an active enrollment status, who are residing in Sabah. As a result of the national lockdown during COVID-19, the survey was distributed virtually using social media platforms such as *Facebook* and *Whatsapp* using the *KoBoToolbox* medium. The survey was conducted from 21 October 2021 to 6 December 2021, equivalent to 47 days.

### 2.3. Questionnaire Design

The questionnaire in the survey is divided into Section A and Section B. The questionnaire in Section A focuses on the demographic characteristics of respondents, whereas Section B concentrates on the challenges of virtual learning among students. There are 35 questions in Section B. All of the questions are measured using the Likert scale with 5 choices, with 1 equivalent to strongly disagree, and 5 equivalents to strongly agree. The questions in Section B are illustrated in the negative, while the remaining questions are adapted from previous studies [38,39,40].

### 2.4. Questionnaire Validity and Reliability

This study begins with a pilot study to ensure the validity and reliability of the instruments that will be used in the survey. According to Johanson and Brooks [41], the reliability of the survey instrument can be assessed using the Cronbach Alpha with 30 respondents for a minimum sampling. The pilot study for the survey consisted of 50 respondents, exceeding the required minimum sampling.

The results of the validity test in this study show that all the variables in construct B are valid due to having a correlation coefficient value (rᵪᵧ) that is greater than the critical value for Pearson’s Correlation coefficient r [42]. To measure validity, this study used the critical value of the Pearson’s Correlation coefficient r with a significance level of 0.5%, which is only 0.273 [43]. This is proven when the minimum value of the correlation coefficient for variables in construct B shows a value of 0.299. The results of the reliability test show that the Cronbach alpha value for construct B can be classified as excellent, which is 0.935 [44]. All these indicators of the questionnaire instruments illustrate that the eligibility of this study meets the requirements of validity and reliability.

### 2.5. Data Analysis

**Figure 2:**
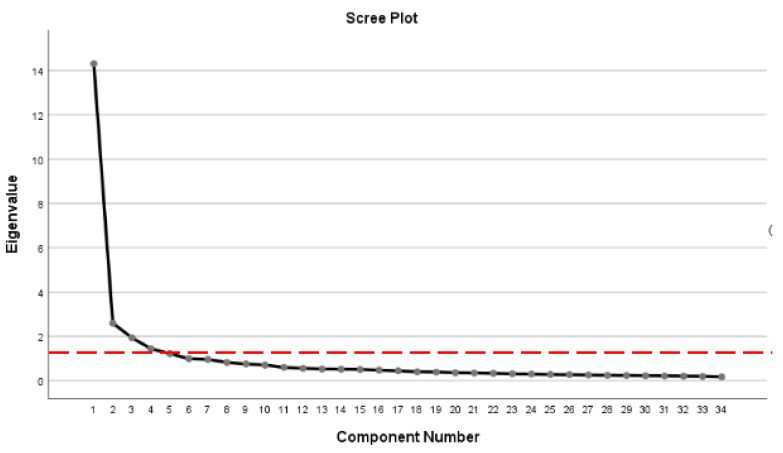
Number of components.

The raw data of this study were analyzed descriptively (frequency, percentage and mean) and inferentially (Principal Component Analysis, K-Means Clustering and Spatial analysis) using the IBM SPSS (Statistical Package for Social Science) version 26. The Principal Component Analysis (PCA) application in this study aimed to simplify the construct B data. The PCA analyzes a data table based on the data observation, which can be described by several inter-correlated quantitative dependent variables. The PCA also assists this study in identifying and extracting any essential information from the statistical data and represent it as a set of new orthogonal variables called principal components [45]. The PCA analysis was carried out twice, since the value of the loading factor (commonality) of the B24 variable, which refers to the inability to focus on virtual learning due to disturbances in the home environment, when carried out the first time was less than 0.5. Hence, before starting the PCA analysis for the second cycle, the B24 variables had to be removed. This is because, according to Kirch et al. [46] and Simanjuntak [47], the eligibility criteria of variables that can be analyzed should begin with a loading factor of 0.5 and above. Barlett’s test result also showed a significant value (x^2^ = 31466.65, df = 561, *p* < 0.05), indicating that the sample deserves further analysis. The number of components was identified through the Scree Plot by considering eigenvalues higher than 1 [48]. This means that the total number of components formed is five (Figure 2).

The cumulative value of the variance for the five components amounts to 63.2% (Table 1). In other words, more than half (63.23%) of the challenges met by higher education students in Sabah are represented by five components, while the rest are caused by other factors [49]. In the humanities field, a cumulative value of variance worth 50% is sufficient for analysis [50].

The five components were then analyzed using K-Means Clustering. This aims to group the sample (five components) into six clusters. This follows the purpose of K-Means Clustering analysis, which is to group data into one group, where the data in one group have distinctive characteristics from the data found in other groups [51]. The K-Means Clustering analysis equation is as follows:

(1) $$J=\overset{k}{\underset{i=1}{\sum }}\overset{n}{\underset{j=1}{\sum }}{\left(x_{i}-v_{j}\right)}^{2}=1$$

where $x_{i}-v_{j}$ is the Euclidean distance between point $x_{i}$ and a centroid, $v_{j}$ iterated over all k points in the $i^{th}$ cluster, for all n clusters. 

**Figure 3:**
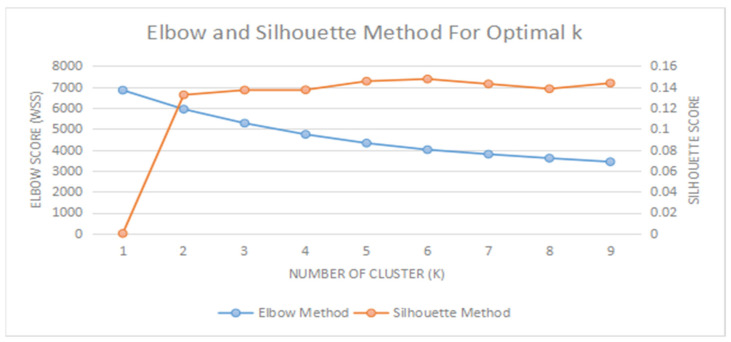
Optimal number of clusters.

Several approaches or methods can be used to determine the optimal number of clusters (*k*), such as through the Average Silhouette Method, Elbow Method, and Gap Statistics [52,53,54],. Nevertheless, the optimal number of clusters (*k*) of this study was determined using the Elbow and Silhouette Methods [53,54]. The elbow and silhouette graphs were produced using machine learning analysis (Jupyter-Anaconda3) and the result demonstrates the optimal number of clusters (k) to be six, as indicated in Figure 3.

To simplify the data interpretation process, the five resulting components are presented using the mean values. The mean values are grouped into five categories, namely, strongly disagree (1.00–1.80), do not agree (1.81–2.0), neutral (2.61–3.40), agree (3.41–4.20), and strongly agree (4.21–5:00) [55,56,57]. This shows that the higher the mean value, the higher the number of respondents who agree with the statement of one component. The last step is to present the cluster results in a mapping system using the Geographic Information System (GIS) application. This mapping process is important in facilitating the identification of clusters that dominate a certain district based on the data.

## 3. Results

### 3.1. Demography

Most of the respondents in this study were single in their marital status (1329 respondents, 96.9%), and a significant majority were studying in public universities (1318 respondents, 95.8%). Based on gender, there were more female respondents (953 persons, 69.5%) compared to male respondents (418 persons, 30.5%). The respondents in this study were also, on average, Muslim Bumiputera (821 persons, 60%). A piece of complete information regarding the demographic background of the respondents is shown in Table 2.

### 3.2. The Challenges of Virtual Learning during COVID-19 in Sabah

Based on the PCA, there are five main challenges faced by students in higher education during the implementation of virtual learning, as shown in Table 3. On average, most of these students stated that virtual learning (P&P) from home is (Co1) not conducive (var(X) = 20.12%). In addition, they also stated that virtual learning from home led to health problems such as (Co2) the deterioration of physical health (var(X) = 13.40%) and (Co3) the decline of mental health (var(X) = 12.10%). The results of this study also show that students face (Co4) limited educational facilities (var(X) = 10.14%) and (Co5) social isolation (var(X) = 7.47%).

### 3.3. Challenges of Virtual Learning Based on Clusters Division

**Figure 4:**
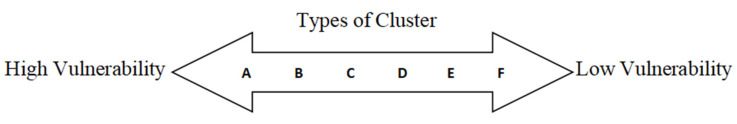
Comparison of the level of vulnerability between each of the clusters.

This study found that there are six clusters of students in Sabah, represented by symbols A, B, C, D, E, and F. The largest number of students is shown in the Cluster A category (21.1%). Students in the cluster category A face all five challenges. Nevertheless, the three most frequently experienced challenges that can be seen in all the six clusters are the following: Co1 (M = 4.39, SD = 0.44), Co2 (M = 4.29, SD = 0.47), and Co3 (M = 4.23, SD = 0.58). This shows that students in the Cluster A category are the most vulnerable, as compared to the other five clusters (Table 4; Figure 3). The total percentage of students in the Cluster C category is the lowest (13.8%). Students in the cluster category A also tend to experience problems with Co1 (M = 3.83, SD = 0.59), Co2 (M = 3.80, SD = 0.62), Co4 (M = 3.55, SD = 0.73) and Co5 (M = 4.01, SD = 0.75). This study also found that students in the Cluster F category have the lowest level of vulnerability during the implementation of virtual learning from home, compared to the other five clusters. Additionally, the average students in the F cluster category do not agree that virtual learning from home causes Co3 (M = 2.45, SD = 0.75) and Co5 (M = 2.54, SD = 0.73). A detailed explanation of the challenges of virtual learning from home based on the division of clusters is shown in Table 4. In addition, Figure 4 also identifies the level of vulnerability based on the ranking of each of the clusters.

### 3.4. The Dominance of Student Clusters in Each District in Sabah

**Figure 5:**
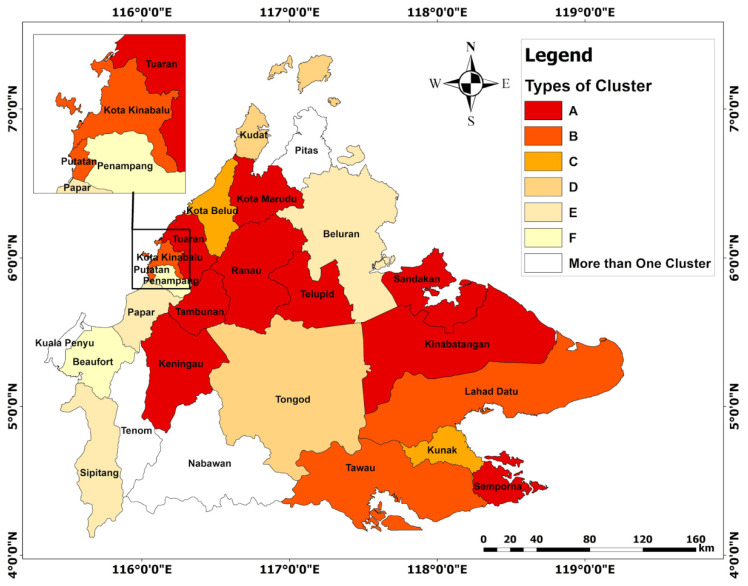
Cluster dominance of each district in Sabah.

The GIS mapping results show that students in Cluster A are in Semporna, Ranau, Sandakan, Kota Marudu, Tuaran, Kinabatangan, Keningau, Sipitang, and Telupid. Students in Cluster B are in Kota Kinabalu, Putatan, Tawau, and Lahad Datu. Students in Cluster C are in Kunak and Kota Belud, Cluster D are in Kudat and Tongod, and Cluster F are in Beaufort and Penampang. The remaining students in Cluster E are in Papar, Beluran, and Tambunan. There is also a hybrid result showing that there is more than one location representing each of the clusters. In these findings based on Figure 5, there is a small percentage of students in Cluster A and D residing in Kuala Penyu and Pitas; some students in Clusters A, B, and C are located in Tenom; and some students in Clusters B, E and F are located in Nabawan.

## 4. Discussion

This study found that there are six clusters in Sabah, each of which deals with several types of challenges, as shown in Figure 3. The findings of this study differ significantly from those of Aboagye [5], who discovered that higher education students in Ghana are currently facing eight main types of challenges (social issues, lecturer issues, accessibility, learner motivation, academic issues, generic issues, learner intentions, and demographics). One of the most typical challenges faced by students in Sabah and Ghana is limited educational facilities (Section 3.2). More than half of the total number of students (51.3%) can be identified in Clusters A, C, and D. In these three clusters, students are identified to face limitations in their educational facilities, as shown in Table 4. Additionally, the media reports indicate that as many as 52% of students in Sabah do not have adequate educational facilities, including internet access and electronic devices, to participate in virtual learning sessions [58,59]. In some severe cases of poor facilities and searching for internet coverage, some of these students had to climb a hill [60], while some of them had to walk up to three kilometers from their homes [61] or visit the nearest decent location, such as a beach [62], to enable them to access the internet. There is even a small percentage of students in the most impoverished districts [63,64] who were unable to access the internet and were almost dismissed from university due to their abstinence from participating in virtual learning [65].

This demonstrates that students’ limited access to educational facilities (poor internet and electronic device accessibility) is the greatest barrier to their capacity to utilize virtual learning. The results of this study are consistent with those of Aboagye [5] in Ghana and Ahmed and Nwagwu [66] in Africa, who discovered that students in developing countries have the greatest problems when virtual learning is adopted due to limited access to educational resources. However, this contradicts the results of Muilenberg and Berge [67] in their study in developed countries, as they found that costs and internet connection are less significant barriers to the application of virtual learning. This demonstrates that geographical factors, whether on a global or local scale, have a considerable effect on the challenges students face while implementing virtual learning.

Therefore, in the context of this study, to deal with the lack of educational facilities, empowerment measures should be concentrated in the 15 districts that are considered rural areas in Sabah. These 15 districts are as follows: Semporna, Ranau, Sandakan, Kota Marudu, Tuaran, Kinabatangan, Keningau, Sipitang, Telupid, Kunak, Kota Belud, Kudat, Tongod, Kuala Penyu and Tenom (Section 3.4). Limited educational facilities in rural areas in Malaysia [68,69], especially in Sabah [70], is not something foreign, as this condition is also prevalent in most other developing countries, such as India [71], Nigeria [6] and China [34]. Therefore, empowerment measures should be improved through civil societies and governmental assistance. Existing governmental assistance to increase the ownership of computer devices occurs through the Free Tablet Subsidy Scheme [72], the Free 1 Malaysia Netbook Programme [73], and the 2022 PerantiSiswa Programme 2022 [74]. This assistance should be extended and enhanced in the 15 rural districts of Sabah. 

The impact of virtual learning on the deterioration of physical health, such as is experienced by students in Clusters A, B, C, and E, should also not be taken lightly, as the percentage of students in these clusters is large (67%). The number of those facing physical health problems also exceeds the number of students who suffered from mental health disorders (Section 3.3). However, the concern over mental health is also alarming, as students in higher education in Malaysia were reported to be severely affected by COVID-19. They have been facing complications of mental health, such as stress, depression and anxiety, during the COVID-19 pandemic [75]. On a different note, the deterioration of physical health is also caused by the prolonged use of electronic devices, such as computers, tablets, and smartphones, that expose students to further radiation released from the screen [76,77], especially during the afternoon virtual learning session [78]. In addition to the prolonged use of electronic devices, other physical health problems occurred, such as major vision problems including eye strain, red eye, dried eyes [76], itchiness in vision, bleary eyes and seeing double, and other related risks such as headaches [79]. Excessive exposure to radiation over an extended period also risks causing cancer and tumors [78]. Other physical health problems caused by the prolonged use of electronic devices include skeleton pain, and muscle problems such as neck pain [80,81], back pain, and shoulder pain [82]. Therefore, several preventive measures can be taken through personal efforts to reduce the risk of health problems, such as avoiding the prolonged use of electronic devices, frequent conscious blinking, using matte or non-reflective screens on electronic devices, and doing light exercises on some parts of the body, such as wrists, hands, neck, arms, legs, and shoulders [79]. To reduce these problems, the focus of empowerment should be placed on vulnerable students who predominantly reside in the 21 districts (refer to Section 3.4). 

In addition, more than half (67.7%) of the students (Section 3.3) stated that virtual learning activities are not conducive (Section 3.4). All of these students reside in various districts in Sabah (Semporna, Ranau, Sandakan, Kota Marudu, Tuaran, Kinabatangan, Keningau, Sipitang, Telupid, Kota Kinabalu, putatan, Tawau, Lahad Datu, Kunak, Kota Belud, Kudat, Tongod, Kuala Penyu, Pitas, Tenom and Nabawan). This finding agrees with the study of Azlan et al. [83], which found that virtual learning limits communication with lecturers and tends to be dull in its implementation, and thus reduces the capacity to focus while learning among students. In Sabah, poor digital literacy also further complicates the issues related to the virtual learning environment (Section 3.4). Limited educational resources and poor facilities in Sabah have led to poor digital literacy among the population in the state [58,59]. 

As such, the existing circumstances of limited access to educational facilities will certainly hinder digital literacy among students in Sabah, as evidenced by the result of this study, with 76% of students thinking that virtual learning is not conducive belonging to the same category as the students with limited educational facilities (Table 4).

## 5. Conclusions

In conclusion, there are six clusters of students in Sabah (Clusters A, B, C, D, E and F), with each of these clusters facing different challenges in participating in virtual learning. Based on the assessment of location, almost half of the districts in Sabah are dominated by students from Cluster A with a total of nine districts, and Cluster B with four districts. Both clusters can be considered highly vulnerable groups in the context of virtual learning. This shows that the empowerment of vulnerable groups among students in Sabah should be addressed immediately in tandem with the fourth goal of the SGD, which is to provide inclusive and high-quality education for all groups. In addition, it can be inferred that locational disparities in developing countries significantly impact the types of challenges that higher education students encounter while using virtual learning. The distinction in location refers to a micro area that encompasses a district or region. In other words, the degree of susceptibility of the students residing in a state (macro) in a developing nation varies, and cannot be generalised. This is because it is quite probable that the challenges experienced by students in the same district and state differ from those described in this study.

Hence, it is hoped that the results of this study could inform the relevant authorities, including policymakers and stakeholders, on the empowerment process of virtual learning, so that it can be delivered more effectively and systematically, whether at the macro or the micro level. In the context of state management (referring to the macro level), the priorities of empowerment should be targeted at the most vulnerable districts, based on the level of vulnerability demonstrated in this study. Meanwhile, at the district management level (referring to the micro level), local authorities should identify the challenges in their respective districts to help higher levels, such as state management, with the strategies for empowerment and improvement that can be carried out in their districts. Similar empowerment initiatives are relevant not just to Sabah and Malaysia, but also to other developing nations. Apart from that, this study contributes to the existing literature on the challenges met during virtual learning, especially in the most impoverished state in Malaysia, Sabah. Undoubtedly, existing studies have highlighted the challenges of virtual learning in Malaysia and other countries. Nevertheless, existing studies do not address the dynamics of location, and the challenges each of these locations faces. Moreover, there is a lack of studies using the methodology of mapping as an instrument to understand the challenges in virtual learning, as this study has done. To conclude, this study fills the current gap in previous studies by assessing the challenges of virtual learning faced by students based on differences in their residential location using geographical mapping analysis.

## Figures and Tables

**Table 1:** Cumulative values of variance for five components.

Component	Initial Eigenvalues		
	Total	% Variance	Cumulative %
1	14.31	42.09	42.09
2	2.60	7.63	49.72
3	1.94	5.70	55.42
4	1.44	4.24	59.65
5	1.22	3.58	63.23
6–24	0.99–0.17	2.90–0.50	66.13–100

**Table 2:** Demographic profile of respondents.

Characteristics	Category	Frequency	Percent (%)
**Gender**	Male	418	30.5
	Female	953	69.5
**Marital Status**	Single	1329	96.9
	Married	42	3.1
**Ethnic**	Muslim Bumiputera	821	60
	Non-Muslim	491	36
	Non-Bumiputera	59	4
**Institutional Status**	Public university	1313	95.8
	Private university	58	4.2

**Table 3:** Analysis results of main component extraction.

Component (Domain)/Item	Loading Factor	Variance (%)
***Component 1 (Co1) Unconducive learning***		
(B21) Difficulty concentrating due to tedious virtual learning methods	0.765	20.12
(B22) Likely to feel bored due to limited virtual learning activities	0.750	
(B17) Difficulty in understanding the contents of the subject taught	0.729	
(B18) Decline of learning productivity	0.686	
(B20) Difficulty in completing group assignments digitally	0.676	
(B34) Difficulty in becoming proficient in virtual learning	0.670	
(B33) I found virtual learning is difficult to implement/follow	0.667	
(B35) Using virtual learning is not as easy as conventional physical learning	0.627	
(B15) Liable to sleepiness during virtual class	0.615	
(B16) Lack of motivation as the learning environment at home is not the same as the learning environment at university	0.606	
(B8) Lack of motivation due to poor communication through face-to-face interaction with peers and lecturers	0.561	
(B23) Difficulty focusing due to in conducive environment at home	0.538	
(B19) Feeling overloaded with university tasks	0.515	
***Component 2 (Co2) Deterioration of physical health***		
(B4) Back pain	0.795	13.40
(B3) Neck pain	0.770	
(B6) Eye fatigue	0.753	
(B2) Headache	0.681	
(B5) Blurry vision	0.680	
(B7) Extreme fatigue	0.600	
(B1) Insomnia	0.515	
***Component 3 (Co3) Decline of mental health***		
(B12) Likely to feel depressed (depression)	0.784	12.10
(B13) Likely to feel stressed (stress)	0.719	
(B14) Likely to feel anxious (anxiety)	0.691	
(B10) Feeling isolated	0.657	
(B11) Lack of personal/physical attention	0.656	
(B9) Feeling lonely	0.614	
***Component 4 (Co4) Limited educational facilities***		
(B29) My internet access is limited due to the low internet network in my home area	0.739	10.14
(B30) Frequent power outages at my home	0.689	
(B31) My laptop has a slow technical performance	0.679	
(B28) My internet access is limited due to the high internet cost	0.666	
(B32) I had to share a personal computer (laptop) with my siblings	0.629	
***Component 5 (Co5) Social isolation***		
(B25) Poor relationship with coursemates	0.740	7.47
(B27) Lack of interpersonal relationships with peers in university	0.713	
(B26) Difficulty communicating with peers online	0.637	

**Table 4:** Challenges of virtual learning based on clusters.

Component Cluster (C)		C01	Co2	Co3	Co4	Co5	n	%
**A**	Mean (M)	4.39	4.29	4.23	4.06	4.07	289	21.1
	Standard Deviation (SD)	0.44	0.47	0.58	0.58	0.79		
**B**	Mean (M)	3.96	3.95	3.67	2.44	4.05	223	16.3
	Standard Deviation (SD)	0.51	0.58	0.67	0.63	0.75		
**C**	Mean (M)	3.83	3.80	2.52	3.55	4.01	190	13.8
	Standard Deviation (SD)	0.59	0.62	0.77	0.73	0.75		
**D**	Mean (M)	3.59	3.11	3.31	3.50	4.11	225	16.4
	Standard Deviation (SD)	0.58	0.65	0.73	0.68	0.60		
**E**	Mean (M)	2.86	3.89	3.06	3.02	3.07	216	15.8
	Standard Deviation (SD)	0.61	0.61	0.90	0.74	0.89		
**F**	Mean (M)	2.98	2.71	2.45	2.68	2.54	228	16.6
	Standard Deviation (SD)	0.75	0.69	0.75	0.80	0.73		

Mean Value: 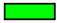 strongly disagree (1.00–1.80); 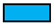 do not agree (1.81–2.60); 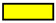 neutral (2.61–3.40); 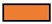 agree (3.41–4.20); 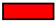 strongly agree (4.21–5.00).

## Data Availability

All data generated or analyzed during this study are included in this published article (and its Supplementary Information Files).

## Supplementary Materials

The following supporting information can be downloaded at: https://www.mdpi.com/article/10.3390/ijerph191711108/s1.

## References

[1] Malaysia Sustainable Development Goals (SDG) Indicators. https://www.Epu.Gov.My/Sites/Default/Files/2021-02/Sustainable-Development-Goals-%28Sdg%29-Indicators-Malaysia-2019.Pdf.

[2] Shava G.N., Chasara T., Hahlani O.S. (2021). Sustainable Development Goal (SDG) 4 on quality in education, Current Issues in Zimbabwe Higher Education, Educating for the future. Int. J. Innov. Soc. Sci.

[3] Ohalezim N.B., Edwards B.I., Aderemi T.J. (2021). Future of Special Education: Options for Equitable eLearning Opportunities for Learners with Special Education Needs. Emerging Technologies for Next Generation Learning Spaces.

[4] Boeren E. (2019). Understanding Sustainable Development Goal (SDG) 4 on “quality education” from micro, meso and macro perspectives. Int. Rev. Educ..

[5] Aboagye E., Yawson J.A., Appiah K.N. (2020). COVID-19 and E-learning: The challenges of students in tertiary institutions. Soc. Educ. Res..

[6] Adeoye I.A., Adanikin A.F., Adanikin A. (2020). COVID-19 and e-learning: Nigeria tertiary education system experience. Int. J. Res. Inn. Appl. Sci..

[7] Mathrani A., Sarvesh T., Umer R. (2021). Digital divide framework: Online learning in developing countries during the COVID-19 lockdown. Glob. Soc. Educ..

[8] Jafar A., Geogre F., Mapa M.T., Sakke N., Dollah R. (2021). Perceptions of urban poor with B40 status on the impact of the implementation of movement control order (MCO) by employment sector: A case study of Kota Kinabalu City, Sabah. J. Contemp. Issues Bus. Gov..

[9] Jafar A., Mapa M.T., Sakke N., Dollah R., Joko E.P., Atang C., Awang Ahmad S., Vun Hung C., Geogre F. (2022). Vaccine hesitancy in East Malaysia (Sabah): A survey of the national COVID-19 immunisation programme. Geospat. Health.

[10] Jafar A., George F., Meri A., Chong V.H., Mapa M.T., Sakke N., Atang C., Dollah R., Joko E.P., Baco Z. (2021). Keberkesanan Program Imunisasi COVID-19 Kebangsaan di Malaysia Timur. Malays. J. Soc. Sci. Humanit..

[11] Jafar A., Dambul R., Dollah R., Sakke N., Mapa M.T., Joko E.P. (2022). COVID-19 vaccine hesitancy in Malaysia: Exploring factors and identifying highly vulnerable groups. PLoS ONE.

[12] Dollah R., Jafar A., Joko E.P., Sakke S., Mapa M.T., Atang C., Hung C.V., George F. (2022). Perception of youth in East Malaysia (Sabah) towards the Malaysia national covid-19 immunisation programme (PICK). J. Public Health Dev..

[13] Peters D., Omar M.O., Dollah R., Wan Hassan W.H. (2022). Undocumented Workers during Malaysia’s Movement Control Order (MCO). Migr. Lett..

[14] United Nation (2022). Economic and Social Council. https://sustainabledevelopment.un.org/content/documents/29858SG_SDG_Progress_Report_2022.pdf..

[15] Keržič D., Alex J.K., Pamela Balbontín Alvarado R., Bezerra D.d.S., Cheraghi M., Dobrowolska B., Fagbamigbe A.F., Faris M.E., França T., González-Fernández B. (2021). Academic student satisfaction and perceived performance in the e-learning environment during the COVID-19 pandemic: Evidence across ten countries. PLoS ONE.

[16] Zou C., Li P., Jin L. (2021). Online college English education in Wuhan against the COVID-19 pandemic: Student and teacher readiness, challenges and implications. PLoS ONE.

[17] Ating R. Challenges to Learning and Teaching in Malaysia in the Time of COVID-19. https://shapesea.com/op-ed/covid-19/challenges-to-learning-and-teaching-in-malaysia-in-the-time-of-covid-19/.

[18] Loganathan T., Chan Z.X., Hassan F., Kunpeuk W., Suphanchaimat R., Yi H., Majid H.A. (2021). Education for non-citizen children in Malaysia during the COVID-19 pandemic: A qualitative study. PLoS ONE.

[19] Muthuprasad T., Aiswarya S., Aditya K.S., Jha G.K. (2021). Students’ perception and preference for online education in India during COVID-19 pandemic. Soc. Sci. Humanit. Open.

[20] Yeo S.C., Lai C.K., Tan J., Gooley J.J. (2021). A targeted e-learning approach for keeping universities open during the COVID-19 pandemic while reducing student physical interactions. PLoS ONE.

[21] Crawford J., Butler-Henderson K., Rudolph J., Malkawi B., Glowatz M., Burton R., Magni P.A., Lam S. (2020). COVID-19: 20 countries’ higher education intra-period digital pedagogy responses. J. Appl. Learn. Teach..

[22] Day M. (2020). COVID-19: Surge in cases in Italy and South Korea makes pandemic look more likely. BMJ.

[23] Agormedah E.K., Henaku E.A., Ayite D.M.K., Ansah E.A. (2020). Online learning in higher education during COVID-19 pandemic: A case of Ghana. J. Educ. Technol. Online Learn..

[24] Zalat M.M., Hamed M.S., Bolbol S.A. (2021). The experiences, challenges, and acceptance of e-learning as a tool for teaching during the COVID-19 pandemic among university medical staff. PLoS ONE.

[25] Fawaz M., Samaha A. (2021). E-learning: Depression, anxiety, and stress symptomatology among Lebanese university students during COVID-19 quarantine. Nurs. Forum..

[26] IAU The Impact of COVID-19 on Higher Education Worldwide Resources for Higher Education Institutions. https://www.iauaiu.net/IMG/pdf/COVID-19_and_he_resources.pdf.

[27] Kabir H., Nasrullah S.M., Hasan M.K., Ahmed S., Hawlader M.D.H., Mitra D.K. (2021). Perceived e-learning stress as an independent predictor of e-learning readiness: Results from a nationwide survey in Bangladesh. PLoS ONE.

[28] Kamsani I.I., Mahat A. (2021). COVID-19: Impak e-pembelajaran terhadap kesihatan pelajar universiti (Covid-19: The impact of e-learning on the health of university students). J. Dunia. Pendidik..

[29] Kecojevic A., Basch C.H., Sullivan M., Davi N.K. (2020). The impact of the COVID-19 epidemic on mental health of undergraduate students in New Jersey, cross-sectional study. PLoS ONE.

[30] Marelli S., Castelnuovo A., Somma A., Castronovo V., Mombelli S., Bottoni D., Leitner C., Fossati A., Ferini-Strambi L. (2020). Impact of COVID-19 lockdown on sleep quality in university students and administration staff. J. Neurol..

[31] Abbasi S., Ayoob T., Malik A., Memon S.I. (2020). Perceptions of students regarding e-learning during covid-19 at a private medical college. Pak. J. Med. Sci..

[32] John Lemay D., Doleck T., Bazelais P. (2021). Transition to online teaching during the COVID-19 pandemic. Interact. Learn. Environ..

[33] Aucejo E.M., French J., Araya M.P.U., Zafar B. (2020). The impact of COVID-19 on student experiences and expectations: Evidence from a survey. J. Public Econ..

[34] Zhu Y., Zhang J.H., Au W., Yates G. (2020). University students’ online learning attitudes and continuous intention to undertake online courses: A self-regulated learning perspective. Educ. Technol. Res. Dev..

[35] Borneo Today Guru Luar Bandar, Pedalaman Sabah Berdepan Cabaran Pastikan Kelancaran Pdpc Dalam Talian. https://www.Borneotoday.Net/Guru-Luar-Bandar-Pedalaman-Sabah-Berdepan-Cabaran-Pastikan-Kelancaran-Pdpc-Dalam-Talian/.

[36] Hamzah I.N.S., Mahamod Z. (2021). Online Teaching strategies used by Malay Language Teachers in improving reading primary school students. J. Pendidik. Bhs. Melayu.

[37] Adam A.M. (2020). Sample size determination in survey research. J. Sci. Res. Rep..

[38] Kim K.J., Liu S., Bonk C.J. (2005). Online MBA students’ perceptions of online learning: Benefits, challenges, and suggestions. Internet High Educ..

[39] Zembylas M., Theodorou M., Pavlakis A. (2008). The role of emotions in the experience of online learning: Challenges and opportunities. Educ. Media Int..

[40] Adnan M., Anwar K. (2020). Online Learning amid the COVID-19 Pandemic: Students’ Perspectives. Online Submiss..

[41] Johanson G., Brooks G. (2010). Initial Scale Development: Sample Size for Pilot Studies. Educational and Psychological Measurement. Educ. Psychol. Meas..

[42] Fanani I., Djati S.P. (2016). The Influence of Job Satisfaction and Organizational Commitment on Organizational Citizenship Behavior (Pengaruh Kepuasan Kerja dan Komitmen Organisasi terhadap Organizational Citizenship Behavior (OCB)). Fundam. Manag. J..

[43] Niño-Zarazúa M. (2012). Quantitative Analysis in Social Sciences: An Brief Introduction for Non-Economists.

[44] Taber K.S. (2018). The use of Cronbach’s alpha when developing and reporting research instruments in science education. Res. Sci. Educ..

[45] Mishra S., Sarkar U., Taraphder S., Datta S., Swain D., Saikhom R., Panda S., Laishram M. (2017). Multivariate statistical data analysis-principal component analysis (PCA). Int. J. Livestock Res..

[46] Kirch J.L., Hongyu K., Silva F.D.L., Dias C.T.D.S. (2017). Factorial analysis for the evaluation of satisfaction questionnaires of the statistics course of a federal institution. Es Eng. Sci..

[47] Simanjuntak I.M. (2017). Use of Principal Component Analysis (PCA) Method to Reduce Factors Affecting Coronary Heart Disease in Hospitals. H. Adam Malik Medan Tahun. http://repositori.usu.ac.id/bitstream/handle/123456789/11234/141000025.pdf?sequence=1&isAllowed=y.

[48] De Barros Ahrens R., da Silva Lirani L., de Francisco A.C. (2020). Construct validity and reliability of the work environment assessment instrument WE-10. Int. J. Environ. Res. Public Health.

[49] Nasution M.Z. (2019). Penerapan Principal Component Analysis (PCA) dalam Penentuan Faktor Dominan yang Mempengaruhi Prestasi Belajar Siswa (Studi Kasus: SMK Raksana 2 Medan). J. Teknol. Inf..

[50] Williams B., Onsman A., Brown T. (2010). Exploratory factor analysis: A five-step guide for novices. Austral. J. Paramed..

[51] Helilintar R., Farida I.N. (2018). Penerapan Algoritma K-Means Clustering Untuk Prediksi Prestasi Nilai Akademik Mahasiwa (Application of K-Means Clustering Algorithm for Prediction of Student Academic Value Performance). J. Sains Dan Inform..

[52] Nanjundan S., Sankaran S., Arjun C.R., Anand G.P. (2019). Identifying the Number of Clusters for K-Means: A Hypersphere Density based Approach. arXiv.

[53] Chandu V. (2020). Identification of spatial variations in COVID-19 epidemiological data using K-Means clustering algorithm: A global perspective. MedRxiv.

[54] Abdullah D., Susilo S., Ahmar A.S., Rusli R., Hidayat R. (2022). The application of K-means clustering for province clustering in Indonesia of the risk of the COVID-19 pandemic based on COVID-19 data. Qual. Quant..

[55] Khampirat B. (2020). The relationship between paternal education, self-esteem, resilience, future orientation, and career aspirations. PLoS ONE.

[56] Beyene G. (2021). The Effect of Customer Based Brand Equity on Customer Retention in the Case of BGI Ethiopia. Ph.D. Dissertation.

[57] Kunchai J., Chonsalasin D., Khampirat B. (2021). Psychometric properties and a multiple indicators multiple cause model of the career aspiration scale with college students of rural Thailand. Sustainability.

[58] Selvanathan M., Hussin N.A.M., Azazi N.A.N. (2020). Students learning experiences during COVID-19: Work from home period in Malaysian Higher Learning Institutions. Teach. Public Adm..

[59] BH Online 52 Peratus Pelajar Sabah Tiada Akses Internet. https://www.bharian.com.my/berita/nasional/2020/05/686499/52-peratus-pelajar-sabah-tiada-akses-internet.

[60] Siti Shamila Che Zahari Masalah Capaian Internet Masih Hantui Pelajar. https://www.sinarharian.com.my/article/146951/SUARA-SINAR/Analisis-Sinar/Masalah-capaian-internet-masih-hantui-pelajar.

[61] Astif Ums A. Masalah Internet di Kampung Halaman, Pelajar Kembali ke Kampus. https://demisabah.com/masalah-internet-di-kampung-halaman-pelajar-kembali-ke-kampus/.

[62] Nur Fazlizai Ali PdPR: Pelajar Terpaksa Ikuti Kelas di Bangsal Usang Kawasan Ladang. https://www.astroawani.com/berita-malaysia/pdpr-pelajar-terpaksa-ikuti-kelas-di-bangsal-usang-kawasan-ladang-281014.

[63] Dollah R., Joko E.P., Maraining A., Sakke N. (2018). Nota editor jemputan: Notes from the guest editors. J. Kinabalu.

[64] Joko E.P., Gapar M.H.A., Annuar S.N.S. (2020). Pangrok Sulap: Sedekad memetakan masa lalu, megukirkan masa hadapan. J. Kinabalu.

[65] Abdullah R. PdPR: Internet Lemah, Pelajar TIDAK pernah Sertai Kelas. https://www.astroawani.com/berita-malaysia/pdpr-internet-lemah-pelajar-tidak-pernah-sertai-kelas-280871.

[66] Ahmed A., Nwagwu W. (2006). Challenges and opportunities of e-learning networks in Africa. Development.

[67] Muilenburg L.Y., Berge Z.L. (2005). Student barriers to online learning: A factor analytic study. Distance Educ..

[68] Sua T.Y. (2012). Democratization of secondary education in Malaysia: Emerging problems and challenges of educational reform. Int. J. Educ. Dev..

[69] Hasin I., Nasir M.K.M. (2021). The Effectiveness of the Use of Information and Communication Technology (ICT) in Rural Secondary Schools in Malaysia. J. Educ. e-Learn. Res..

[70] Wahab H.A., Bunyau W., Rezaul Islam M. (2018). Microcredit for rural poverty alleviation and social well-being: A study of Sabah, Malaysia. Asian Soc. Work. Policy Rev..

[71] Roy N.K. (2012). ICT-enabled rural education in India. Int. J. Inf. Educ. Technol..

[72] Majalahsinar Terkini! Pemberian Telefon Bimbit Percuma Kepada Pelajar (Latest! Free Mobile Phones for Students). https://majalahsinar.com/?p=1312.

[73] SarawakVoice PdPR: Beri Gajet Percuma Kepada Pelajar (PdPR: Give Free Gadgets to Students). https://sarawakvoice.com/2021/01/22/pdpr-beri-gajet-percuma-kepada-pelajar/.

[74] Bernama Program PerantiSiswa Keluarga Malaysia Wajar Diperluas ke Peringkat Sekolah (The PerantiSiswa Keluarga Malaysia programme should be explanded to school level). https://www.astroawani.com/berita-malaysia/program-perantisiswa-keluarga-malaysia-wajar-diperluas-ke-peringkat-sekolah-357514.

[75] Moy F.M., Ng Y.H. (2021). Perception towards E-learning and COVID-19 on the mental health status of university students in Malaysia. Sci. Prog..

[76] Poudel S. (2018). A research report about effect of display gadgets on eyesight quality (computer vision syndrome) Of, M. Sc.(CSIT) students in Tribhuvan University. Int. J. Sci. Eng. Res..

[77] Usikalu M.R., Babarimisa I.O., Akinwumi S.A., Akinyemi M.L., Adagunodo T.A., Ayara W.A. (2018). Radiation from Visual Display Unit. IOP Conf. Ser. Earth Environ. Sci..

[78] Bamikole J.A., Musa M.A. (2017). Analysis of nuclear radiations from commonly used electronic gadgets in Lafia, Nigeria. Phys. Sci..

[79] Gunduz S. Health Problems with the Use of Information Technologies. Proceedings of the International Educational Technology (IETC) Conference 7th.

[80] Bugün İ., Yardımcı H., Ertemel S., Öğün A.M., Dinçses E. (2006). Üniversite öğrencilerinin bilgisayar kullanımına ilişkin bilgi, davranış ve ilişkili sağlık sorunları.

[81] Agarwal A., Sharma S., Kumar V., Kaur M. (2021). Effect of E-learning on public health and environment during COVID-19 lockdown. Big Data Min. Anal..

[82] Mbaye I., Fall M.C., Sagnon A., Sow M.L. (1998). Survey of pathology associated with the use of video display terminals. Dakar Med..

[83] Azlan C.A., Wong J.H.D., Tan L.K., Huri M.S.N.A.D., Ung N.M., Pallath V., Tan C.P.L., Yeong C.H., Ng K.H. (2020). Teaching and learning of postgraduate medical physics using Internet-based e-learning during the COVID-19 pandemic—A case study from Malaysia. Phys. Med..

